# Enhancing Antiviral Immunity in the Gastrointestinal Epithelium: The Role of Fibroblast–Endothelium Interaction and Melatonin

**DOI:** 10.3390/cells14130990

**Published:** 2025-06-28

**Authors:** Milda Šeškutė, Goda Laucaitytė, Rūta Inčiūraitė, Mantas Malinauskas, Lina Jankauskaitė

**Affiliations:** 1 Department of Pediatrics, Lithuanian University of Health Sciences, 44307 Kaunas, Lithuanialina.jankauskaite@lsmu.lt (L.J.); 2 Institute of Physiology and Pharmacology, Lithuanian University of Health Sciences, 44307 Kaunas, Lithuania; 3 Institute for Digestive Research, Lithuanian University of Health Sciences, 44307 Kaunas, Lithuania

**Keywords:** gastrointestinal co-culture model, antiviral, melatonin, peroxisomes, IFNλ1, IRF3

## Abstract

The gastrointestinal (GI) tract is a major barrier against pathogens, including viruses. The antiviral responses in the GI epithelium have been broadly investigated, but data on the contribution of the stromal cells remain scarce. Melatonin, widely used to treat insomnia, has recently been proposed as an antiviral agent, yet its effect in the GI tract remains poorly understood. We compared the antiviral responses in Caco-2 monocultures and co-cultures with intestinal fibroblasts (HSIFs) and endothelial cells (HUVECs) after stimulation using Poly I:C. We evaluated the apoptosis, proliferation, key antiviral markers (IRF1, IRF3, IFNs, TBK1, STAT3), and mitochondrial and peroxisomal activation with and without melatonin. The Caco-2 cells cultured with the HSIFs and HUVECs demonstrated enhanced proliferation and reduced Poly I:C-induced apoptosis. The co-culture exhibited a more rapid IRF3-IFNλ1 response, higher TBK1 expression, and enhanced peroxisomal activation compared to these properties in the monoculture. Melatonin further reduced apoptosis and modulated organelle-specific antiviral signaling by suppressing peroxisomal activation and promoting mitochondrial activity. Reduced peroxisomal activation was associated with decreased TBK1, IRF3, and IFNλ1 levels and altered STAT3 signaling. These effects were more pronounced when melatonin was applied post-stimulation compared to that under prophylactic use. Fibroblast–endothelial interactions amplify the antiviral responses in the intestinal epithelial cells by activating the TBK1–IRF3–IFNλ1 axis. Melatonin modulates these responses, highlighting its therapeutic potential in viral GI infections.

## 1. Introduction

The human gastrointestinal (GI) tract is not only responsible for the absorption of nutrients but also plays an important role as a site of entry for various pathogens, including viruses, making it one of the major barriers of host protection [1,2]. Upon viral invasion into the GI epithelial cells, viral detection by pattern recognition receptors (such as Toll-like receptor 3 (TLR3)) activates the innate immune response via transcription factors like interferon regulatory factor 3 (IRF3) and IRF1 [2,3]. This leads to the production of type I and III interferons (IFNs), which in turn trigger the JAK/STAT signaling pathway to ensure the primary antiviral response [3,4]. TANK-binding kinase 1 (TBK1), which phosphorylates IRF3, is one of the central regulators of this process and can be activated at organelle-specific sites, including the mitochondria and peroxisomes [5]. However, the dynamics of this signaling cascade in the gut epithelium remain incompletely understood.

Traditionally, GI immune mechanisms have been investigated in epithelial monoculture models in vitro. However, epithelial immune responses can be influenced significantly by neighboring stromal cells such as fibroblasts and endothelial cells. Co-culture models have recently advanced the study of GI immunity by offering a more physiologically relevant setting for investigating cellular crosstalk and host–pathogen interactions more accurately [6]. Research has shown that fibroblasts and endothelial cells can contribute to tissue homeostasis in the gut and can modulate immune signaling, supporting the antiviral responses in epithelial cells [6,7]. These findings highlight the importance of studying these interactions in more complex model systems.

Currently, no specific treatment exists for viral GI infections. Melatonin, a natural hormone best known for its circadian-rhythm-regulating properties and treating sleep disorders [8], has recently gained attention for its broader therapeutic potential, including its role as an antiviral agent [9,10]. Research suggests that melatonin can modulate immune responses and enhance host defense mechanisms [11], potentially through its influence on the mitochondria, which are increasingly recognized for their role in antiviral signaling. However, despite these promising findings, the precise role of melatonin in GI infections remains unclear.

Therefore, in this study, we compared the antiviral response in a GI co-culture model versus that in an epithelial monoculture and analyzed how melatonin treatment or prophylaxis could influence these processes. Our results indicate that interactions with fibroblasts and endothelial cells support epithelial cell proliferation and survival while also enhancing the protection against virus-mimic-induced damage by activating the type III IFN pathways and targeted organelle-based immune responses. Furthermore, melatonin significantly amplified these responses, highlighting its potential as a mucosal immunomodulator.

## 2. Materials and Methods

### 2.1. The Cell Culture

Intestinal epithelial cells (Caco-2, HTB-37, ATCC, UK), human small intestine fibroblasts (HSIF, P10760, Innovative Technologies and Biological Systems, S.L., Derio, Spain), and human umbilical vein endothelial cells (HUVEC, C0035C, Thermo Fisher Scientific, Waltham, MA, USA) were cultured in 150 cm^2^ tissue culture flasks (TPP, Techno Plastic Products AG, Trasadingen, Switzerland) at 37 °C in a humidified incubator with 5% CO_2_. The culture media were replaced every 2–3 days. Each cell type was maintained in its respective growth media (Table 1). The cells were passaged at 70–80% confluency using TrypLE™ Express Enzyme (Gibco, Life Technologies Corporation, Grand Island, NY, USA). Prior to passaging, viable cell numbers were determined using 0.4% Trypan Blue exclusion and a hemocytometer (Gibco, Life Technologies Corporation, Grand Island, NY, USA). All experiments were conducted using cells between passages 7 and 20.

### 2.2. The Development of the Co-Culture GI Model

To establish a viral co-culture model, cells were harvested using TrypLE™ Express Enzyme, counted with a hemocytometer using 0.4% Trypan Blue solution, and then seeded into 24-well tissue culture plates (TPP, Techno Plastic Products AG, Trasadingen, Switzerland). The HSIFs and HUVECs were first mixed at a 3:1 ratio and seeded at a total density of 3 × 10^4^ cells per well in a 1:1 mixture of their respective culture media. Subsequently, the Caco-2 cells (1 × 10^4^ cells per well in MEM) were seeded onto 0.4 µm-pore-size culture inserts (Brand GMBH, Wertheim, Germany), which were placed above the fibroblast–endothelial layer. The co-cultures were incubated at 37 °C in a humidified incubator at 5% CO_2_, with media changes every 2–3 days for 14 days.

**Figure 1:**
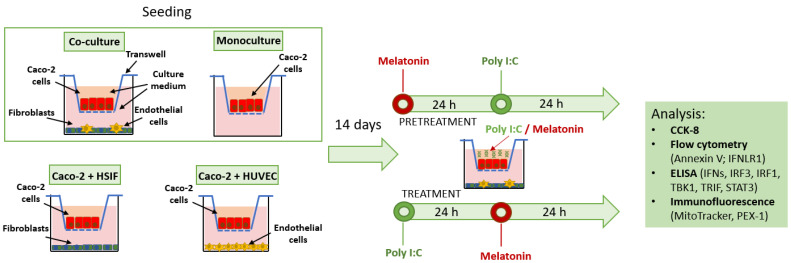
The experimental layout of the co-culture and monoculture models showing the seeding scheme for the Caco-2 cells, HSIFs, and HUVECs into the wells and inserts and the timing of viral mimic stimulation and melatonin treatment. Abbreviations: HSIF–human small intestinal fibroblasts; HUVEC–human umbilical vein endothelial cells; Poly(I:C)–polyinosinic-polycytidylic acid; CCK-8–Cell Counting Kit-8; IFNLR1–interferon-λ receptor 1; IFNs–interferons; IRF3–interferon regulatory factor 3; IRF1–interferon regulatory factor 1; TBK1–TANK-binding kinase 1; TRIF–Toll-like receptor adaptor molecule 1; STAT3–signal transducer and activator of transcription 3; PEX1–peroxisomal biogenesis factor 1; h–hour.

Prior to seeding, the HUVECs were labeled with PKH26 fluorescent membrane dye to facilitate identification via fluorescence microscopy. The presence of HSIF cells in the co-cultures was verified through immunostaining using an anti-α-smooth muscle actin (α-SMA) antibody after fixation, following 14 days of co-culture incubation. In parallel, the Caco-2 monoculture and dual co-cultures (Caco-2 with either HSIFs or HUVECs) were established for the comparative analysis (Figure 1).

### 2.3. Poly I:C Stimulation and Melatonin Treatment

The Caco-2 cells, cultured in transwell inserts for 14 days, were stimulated with the TLR3 agonist polyinosinic–polycytidylic acid (Poly I:C; Tocris Bioscience, Abingdon, UK) at concentrations of 10 μg/mL and 100 μg/mL for 24 h to mimic virus-induced epithelial damage (Figure 1). These concentrations were selected based on our previous findings [12], where Poly I:C induced a dose-dependent inflammatory and barrier-disruptive response without overt cytotoxicity. In the preliminary monoculture experiments, 1 µg/mL showed minimal effect, whereas 10 µg/mL and above caused consistent cellular responses. The higher dose (100 µg/mL) was chosen for these experiments based on the observation that the co-culture exhibited higher resistance to Poly I:C-induced stress.

In the experimental setup, cells were either pretreated with melatonin for 24 h prior to Poly I:C stimulation or treated for 24 h following stimulation. Melatonin was applied at a concentration of 50 μM, consistent with our earlier study [12], which demonstrated that melatonin exerted antiviral and barrier-protective effects under similar in vitro conditions, also without signs of toxicity. This concentration also aligns with studies indicating that the melatonin levels in the gastrointestinal tract may be 10–100 times higher than those in the plasma and falls within the pharmacological range commonly used to study antiviral responses in vitro [13,14]. Melatonin (Sigma-Aldrich, St. Louis, MO, USA) was prepared following the manufacturer’s instructions and diluted in the cell-specific culture media as described above.

### 2.4. Immunofluorescence Microscopy and Image Analysis

The cells were washed with phosphate-buffered saline (PBS) and fixed with 4% paraformaldehyde (PFA) for 15 min at room temperature, followed by permeabilization with 0.2% Triton X-100 (Sigma-Aldrich, St. Louis, MO, USA) for 30 min. After blocking with 10% FBS for 1 h, the HSIF cells were incubated overnight at 4 °C with the Alexa Fluor 647-conjugated mouse α-SMA antibody (1:100, Abcam, Cambridge, UK). For peroxisome labeling, the cells were incubated with a rabbit anti-PEX1 primary antibody (Bethyl, Fortis Life Sciences, Boston, MA, USA) overnight at 4 °C, followed by the Alexa Fluor 488-conjugated anti-rabbit IgG secondary antibody (1:500, Invitrogen, Eugene, OR, USA) for 3 h at room temperature. The nuclei were counterstained with DAPI (Sigma-Aldrich). The HUVECs were fluorescently labeled with PKH26 membrane dye (Sigma-Aldrich) prior to seeding, according to the manufacturer’s protocol, and visualized directly without further staining. All staining steps were conducted in the dark.

For mitochondrial imaging, live cells were incubated with MitoTracker™ (200 nM; Invitrogen, Eugene, OR, USA) for 30 min at 37 °C following the experimental treatments. After incubation, the cells were immediately imaged without fixation.

Fluorescent images were acquired using an inverted microscope (Olympus IX2-SP, Olympus Corporation, Tokyo, Japan) equipped with a digital camera (Olympus DP26) and an X-Cite 120Q fluorescence illuminator (Lumen Dynamics, Mississauga, ON, Canada). Images were captured using cellSens software (version 1.16, Olympus, Japan), and the analysis was performed using ImageJ (v1.53k, National Institutes of Health, Bethesda, MD, USA).

For a quantitative analysis of mitochondrial and peroxisomal staining, images were split into individual color channels and converted into grayscale. The background was subtracted to minimize non-specific signals, followed by despeckling and threshold adjustment to enhance mitochondrial visualization. The mean fluorescence intensity was measured within defined regions of interest (ROIs) and reported in arbitrary units. All imaging and quantification steps were performed in triplicate under consistent acquisition settings.

### 2.5. The Cell Viability Assays

Cell viability was evaluated using Cell Counting Kit-8 (WST-8/CCK8; Abcam, Cambridge, UK) 24 h after Poly I:C stimulation or melatonin treatment. Following replacement of the medium, 10 μL of CCK-8 solution was added to each well and incubated for 2 h. The absorbance was measured at a wavelength of 450 nm using a microplate reader (Thermo Fisher Scientific, Waltham, MA, USA). The control conditions were set as 100% viability.

### 2.6. The Enzyme-Linked Immunosorbent Assay (ELISA)

The antiviral response was evaluated by quantifying interferons (IFNλ1, IFNβ, and IFNα) in the cell culture supernatants and key signaling molecules, including interferon regulatory factors IRF1 and IRF3)) and signal transducer and activator of transcription 3 (STAT3), as well as the adaptor protein Toll-like receptor adaptor molecule 1 (TRIF) and the kinase TBK1, in the cell lysates using human enzyme-linked immunosorbent assay (ELISA) kits. The detection limits for the measured analytes are presented in Table 2.

The cell supernatants and cell lysates (prepared in Cell Lysis Buffer II (Invitrogen, Thermo Fisher Scientific, Carlsbad, CA, USA; Bender MedSystems GmbH, Vienna, Austria) following trypsinization) were immediately stored at −80 °C and thawed only once prior to analysis. ELISAs were conducted according to the manufacturers’ protocols. The optical density (OD) values were measured using a Multiscan Go microplate reader (Thermo Fisher, Vantaa, Finland), and the concentrations were calculated from the standard curves.

### 2.7. Flow Cytometry

Cell apoptosis and the expression of interferon lambda receptor 1 (IFNLR1) were analyzed using multicolor flow cytometry (BD FACSMelody) and BD FACSChorus software (v3.0, Becton, Dickinson and Company, Franklin Lakes, NJ, USA). Following the treatments, the cells (1–5 × 10^5^) were harvested using TrypLE™ Express, washed with PBS, and resuspended in PBS containing 2% FBS.

Apoptosis was assessed using the Annexin V-FITC/propidium iodide (PI) apoptosis detection kit (Invitrogen, Life Technologies Corporation, Eugene, OR, USA). All cells were stained with Annexin V and PI for at least 15 min in the dark at room temperature. IFNLR1 staining was performed according to the manufacturer’s instructions using the primary monoclonal IFNLR1 antibody (Invitrogen, Rockford, IL, USA) and an Alexa Fluor™ 488-conjugated secondary antibody (Invitrogen, Eugene, OR, USA) for at least 60 and 30 min, respectively, at 4 °C. In the co-culture settings, the endothelial cells were pre-labeled with PECAM-1 for 30 min before staining.

Flow cytometry was performed immediately after staining, and the data were analyzed using FlowJo v10.10 software (Becton, Dickinson and Company, Franklin Lakes, NJ, USA). The IFNLR1 expression was quantified according to the median fluorescence intensity (MFI), and the percentage of positive cells was calculated using control-based gating.

### 2.8. The Statistical Analysis

The statistical analysis was performed using IBM SPSS Statistics (v 29.0.2.0, IBM Corp., Armonk, NY, USA) and GraphPad Prism software (version 10.3.1, GraphPad Software, Boston, MA, USA). The data are presented as the mean ± standard deviation (SD). The normality of the data distribution was assessed using the Shapiro–Wilk test. The statistical significance between groups was determined using the unpaired *t*-test for pairwise comparisons of specific conditions. For multiple group comparisons within a single culture model, a one-way ANOVA was performed, followed by Bonferroni’s post hoc test. To determine the combined effects of the culture models and treatment types on the dependent variables, a two-way ANOVA was used, followed by Bonferroni’s post hoc test. Statistical significance was assumed when the *p*-value was less than 0.05 and indicated as * *p* < 0.05, ** *p* < 0.01, and *** *p* < 0.001. The statistical significance of the protein expression over time between the mock- and Poly I:C-stimulated groups was assessed using the Compact Letter Display (CLD) method, where different letters indicate significant differences: lowercase letters denote *p* < 0.05, and uppercase letters denote *p* < 0.01. Correlations were counted using the Pearson’s correlation coefficient. All experiments were performed in triplicate.

Graphs were generated using GraphPad Prism software. Illustrations were created using Microsoft PowerPoint (Office standard 2019, version 1808). AI (ResearchRabbit) was used to identify original articles from the literature to include in the literature review. Open AI (ChatGPT) was used to assist in editing this manuscript in terms of its wording and formatting and to improve the clarity of the text. All AI-generated content was critically reviewed and verified by the author to ensure its accuracy, scientific integrity, and alignment with the current evidence and ethical standards.

## 3. Results

### 3.1. The Development of the Co-Culture GI Model

**Figure 2:**
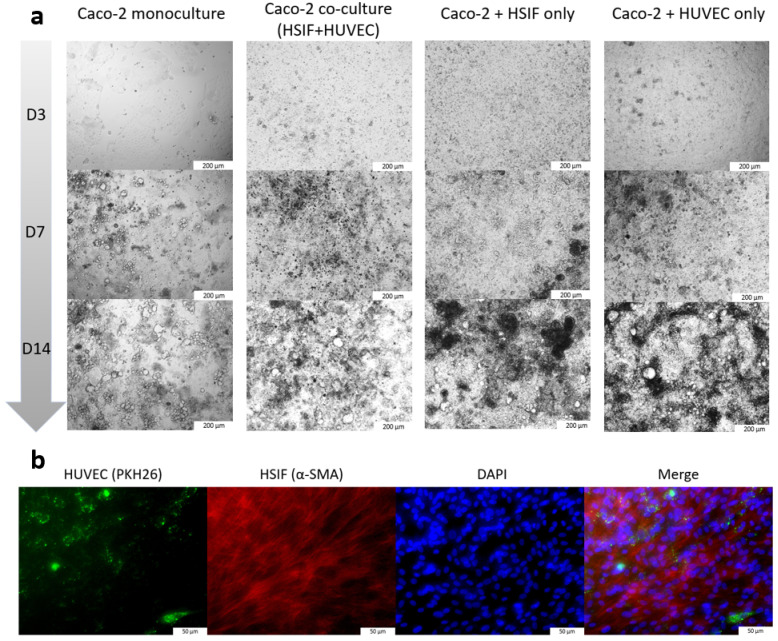
(**a**) Brightfield images showing the growth of the Caco-2 cells in the monoculture or co-cultures with HSIFs and (or) HUVECs from Day 3 to Day 14 (images were acquired at 10× magnification). (**b**) Immunofluorescence images demonstrating the presence of HSIFs and HUVECs in the co-culture model. HUVECs were labeled with PKH26 (green), HSIFs were stained with α-smooth muscle actin (α-SMA; red), and nuclei were counterstained with DAPI (blue) (images were acquired at 20× magnification).

We established a GI co-culture model comprising epithelial (Caco-2) cells, fibroblast (HSIF) cells, and endothelial cells (HUVECs) to evaluate its antiviral response in comparison to that of the Caco-2 monoculture. Brightfield microscopy revealed a slightly faster cell growth rate in the co-culture models compared to that in the monoculture (Figure 2). This observation was corroborated by a 32% increase in the proliferation rate in the Caco-2 cells co-cultured with HSIFs and HUVECs compared to that in the monoculture (1.68 ± 0.224 vs. 1.13 ± 0.029, respectively; *p* = 0.031).

### 3.2. Apoptosis and Viability Following Poly I:C Stimulation

**Figure 3:**
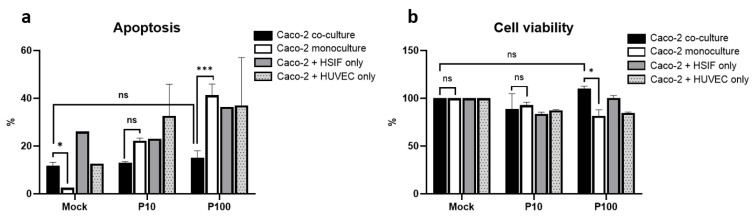
Comparison of cell viability (**a**) and proliferation (**b**) in Caco-2 monoculture and co-culture models after Poly I:C stimulation. ns indicates *p* > 0.05, * indicates *p* < 0.05, and *** indicates *p* < 0.001.

To assess antiviral responses, the models were stimulated with the viral mimic Poly I:C. The effect of Poly I:C depended on the culture model (*p* = 0.028). Poly I:C stimulation induced a dose-dependent increase in apoptosis in the Caco-2 monoculture and in the dual co-cultures with either HSIFs or HUVECs (Figure 3a). After high-dose stimulation (100 μg/mL), the apoptosis levels reached approximately 35–40% in the monoculture and dual co-cultures. In contrast, the triple co-culture exhibited a markedly attenuated apoptotic response, with levels remaining below 20% even at the higher dose (Figure 3a). These results suggest that the combined presence of fibroblasts and endothelial cells provides a protective effect against virus-mimic-induced apoptosis in epithelial cells.

Consistent with the apoptosis data, the Caco-2 cell viability tended to be better preserved in the triple co-culture model compared to that in the monoculture and dual co-cultures (*p* = 0.024) (Figure 3b). High-dose Poly I:C reduced the viability to 76–85% in the latter models, whereas the triple co-culture maintained levels close to those in the mock-treated controls (Figure 3b). Together, these findings support the hypothesis that fibroblast and endothelial support enhances epithelial resistance to viral-mimic-induced cytotoxic stress.

### 3.3. IFN Kinetics and Expression in the Co-Culture

**Figure 4:**
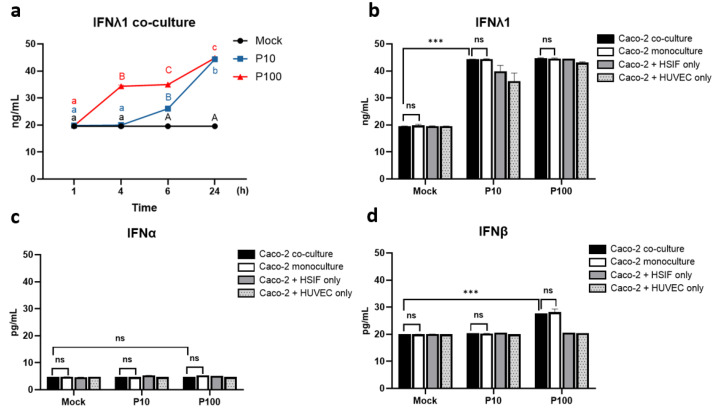
Levels of IFNs measured using ELISA in co-culture and monoculture models 24 h after Poly I:C stimulation: (**a**) levels of IFNλ1 over time in co-culture (different letters indicate significant differences: lowercase letters denote *p* < 0.05, and uppercase letters denote *p* < 0.01); (**b**) IFNλ1 levels; (**c**) IFNα levels; and (**d**) IFNβ levels. ns indicates *p* > 0.05, and *** indicates *p* < 0.001.

We next investigated the type III IFN pathway, which we had previously shown to be critical in GI epithelial cells [12]. The time-course analysis in the triple co-culture model revealed that the IFNλ1 expression increased in a time- and dose-dependent manner (Figure 4a). A higher Poly I:C dose induced earlier IFNλ1 responses, with levels reaching 19.96 ± 0.04 and 26.08 ± 0.08 ng/mL at 4 and 6 h after 10 μg/mL of stimulation and 34.34 ± 0.34 and 34.98 ± 0.02 ng/mL after 100 μg/mL. The mock-treated cells showed no temporal changes (19.57 ± 0.013 ng/mL, *p* < 0.001).

Stimulation with Poly I:C led to a robust increase in the IFNλ1 levels across all models, exceeding 40 ng/mL at both concentrations, with the highest levels in the triple co-culture, though the differences among models were minor (Figure 4b). The mock-treated cells maintained the baseline IFNλ1 levels (20 ng/mL), indicating that Poly I:C was essential to its induction. These findings confirm that all model variants are capable of mounting a strong type III IFN response.

No detectable induction of IFNα was observed under any of the conditions (Figure 4c). Unlike IFNα, IFNβ was modestly induced by high-dose Poly I:C stimulation, particularly in the Caco-2 monoculture (28.18 ± 1.19 pg/mL) and the triple co-culture (27.84 ± 0.23 pg/mL), but not after low-dose stimulation (Figure 4d). This suggests that IFNβ is part of the antiviral response but is induced less robustly than IFNλ1.

### 3.4. Distinct Regulation of IRF3 and IRF1 in the Co-Cultured Caco-2 Cells During the Antiviral Response

**Figure 5:**
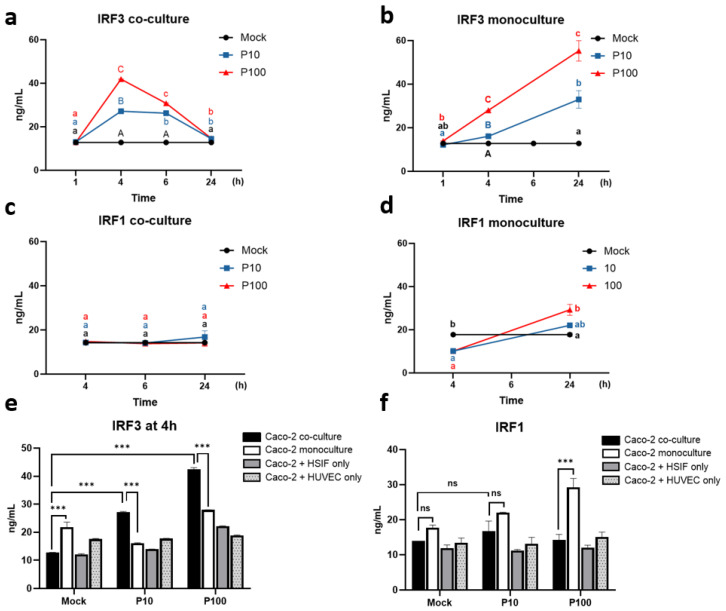
Levels of IRFs measured using ELISA in co-culture and monoculture models after Poly I:C stimulation: IRF3 expression over time in co-culture with HSIFs and HUVECs (**a**) and monoculture (**b**); IRF1 expression over time in co-culture with HSIFs and HUVECs (**c**) and monoculture (**d**); (**e**) IRF3 levels after 4 h of Poly I:C stimulation; (**f**) IRF1 levels after 24 h of Poly I:C stimulation. ns indicates *p* > 0.05, and *** indicates *p* < 0.001. Different letters indicate significant differences: lowercase letters denote *p* < 0.05, and uppercase letters denote *p* < 0.01.

Given the high IFNλ1 levels, we investigated upstream regulators further, including interferon regulatory factors IRF3 and IRF1. The time-course analysis revealed rapid induction of the IRF3 expression in response to Poly I:C, peaking at 4 h in the co-culture (42.45 ± 0.63 ng/mL after high-dose Poly I:C) (Figure 5a) and at 24 h in the monoculture (55.3 ± 3.3 ng/mL) (Figure 5b), indicating faster activation in the co-culture model. In contrast, the IRF1 expression remained relatively stable over time in the co-culture but increased progressively in the monoculture in a dose-dependent manner, peaking at 24 h post-stimulation (Figure 5c,d).

Across all models, IRF3 was upregulated in a dose-dependent manner, with the strongest induction in the triple co-culture (42.45 ± 0.62 ng/mL), compared to that in the monoculture (28.01 ± 0.01 ng/mL) and the dual co-cultures with the HSIFs (22.13 ± 0.18 ng/mL) and HUVECs (18.85 ± 0.21 ng/mL) (*p* < 0.001) (Figure 5e). In contrast, following high-dose Poly I:C, the IRF1 levels in the monoculture were more than double those in the triple co-culture (29.3 ± 2.54 vs. 14.2 ± 1.63 ng/mL, *p* = 0.02) (Figure 5f). This again suggests that the presence of fibroblasts and endothelial cells suppresses IRF1 induction in the epithelial cells, potentially modulating downstream type III IFN signaling.

### 3.5. The TRIF-TBK1 Signaling Axis Is Pre-Activated and Sustained in GI Co-Cultures Following Poly I:C Stimulation

To explore the early innate immune signaling cascade upstream of IRF3 activation, we examined the TBK1 and TRIF dynamics in the monoculture and co-culture models following Poly I:C stimulation.

**Figure 6:**
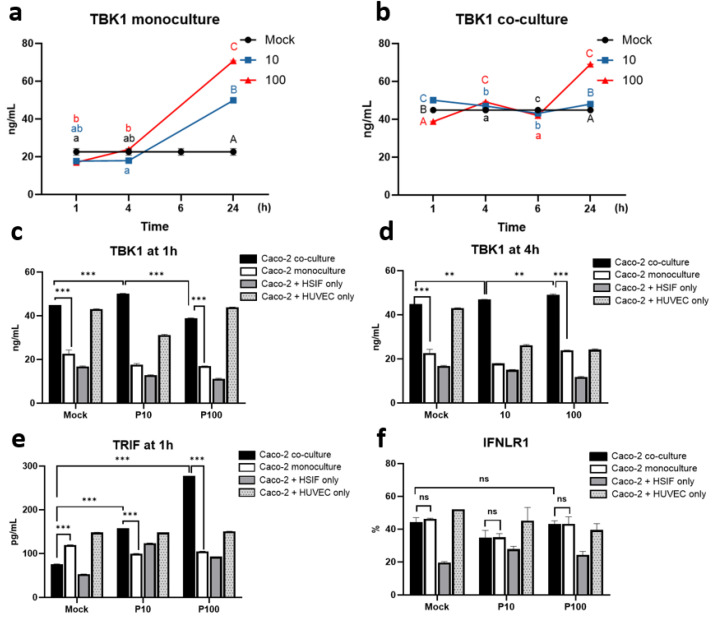
TBK1 expression dynamics in co-cultures compared to monocultures: (**a**) TBK1 expression over time in monoculture; (**b**) TBK1 expression over time in co-culture with HSIFs and HUVECs; (**c**) comparison of TBK1 expression between cultures 1 h after Poly I:C stimulation; (**d**) comparison of TBK1 expression between cultures 4 h after Poly I:C stimulation; (**e**) comparison of TRIF expression between cultures 1 h after Poly I:C stimulation; (**f**) comparison of IFNLR1 expression between cultures 24 h after Poly I:C stimulation using flow cytometry. ns indicates *p* > 0.05, ** indicates *p* < 0.01, and *** indicates *p* < 0.001. Different letters indicate significant differences: lowercase letters denote *p* < 0.05, and uppercase letters denote *p* < 0.01.

The TBK1 expression exhibited dynamic regulation in response to Poly I:C stimulation depending on the culture model (*p* < 0.001) (Figure 6a–d). In the Caco-2 monoculture, the TBK1 levels increased in a dose-dependent manner, peaking at 24 h (70.79 ± 0.2 ng/mL after high-dose Poly I:C) (Figure 6a). In contrast, the triple co-culture exhibited elevated baseline TBK1 levels (45 ng/mL), which remained stable or showed a modest increase over time, indicating a primed activation state (Figure 6b). At early timepoints (1 and 4 h), the TBK1 expression was approximately two-fold higher in the co-culture than that in the monoculture (38.8 ± 0.23 vs. 17.26 ± 0.01 ng/mL at 1 h after 100 µg/mL of Poly I:C, *p* < 0.001) (Figure 6c,d). Notably, elevated TBK1 levels were also seen in mock conditions in the co-culture, suggesting pre-activation via fibroblast and endothelial cell paracrine signaling or cellular crosstalk.

The TRIF expression increased in a dose-dependent manner following Poly I:C stimulation and was significantly higher in the triple co-cultures compared to that in the monoculture (*p* < 0.001) (Figure 6e). TRIF and TBK1 levels were strongly correlated (r = 0.998, *p* < 0.001), supporting coordinated activation of this antiviral axis, potentially contributing to IRF3 activation and downstream IFNλ1 responses.

### 3.6. Sustained IFNLR1 Expression Supports Robust IFNλ1 Signaling in Co-Cultures

To assess whether downstream IFNλ1 signaling was also influenced by receptor availability, we measured the IFNLR1 expression across the models following Poly I:C treatment. Under mock conditions, the IFNLR1 expression was similar in the monoculture (46 ± 0.3%) and the triple co-culture (42.8 ± 2.22%) but significantly lower in the Caco-2 + HSIF co-culture (19.7 ± 0.41%) (*p* < 0.001) (Figure 6f). After Poly I:C stimulation, the IFNLR1 expression slightly decreased across most of the models. However, both the triple co-culture and the monoculture retained relatively high receptor expression (44.1 ± 1.63% and 46.3 ± 3.1%) even after high-dose Poly I:C stimulation (Figure 6f).

These findings suggest that co-culture preserves both the signaling capacity (via TBK1) and responsiveness (via IFNLR1) for type III interferons. Early TBK1 activation and sustained IFNLR1 surface expression in the co-cultures may collectively contribute to a more robust and sustained antiviral state, likely enhancing the efficacy of IFNλ1 responses in the GI epithelium.

### 3.7. Mitochondrial and Peroxisomal Activation Diverges Between Monoculture and Co-Culture Models

**Figure 7:**
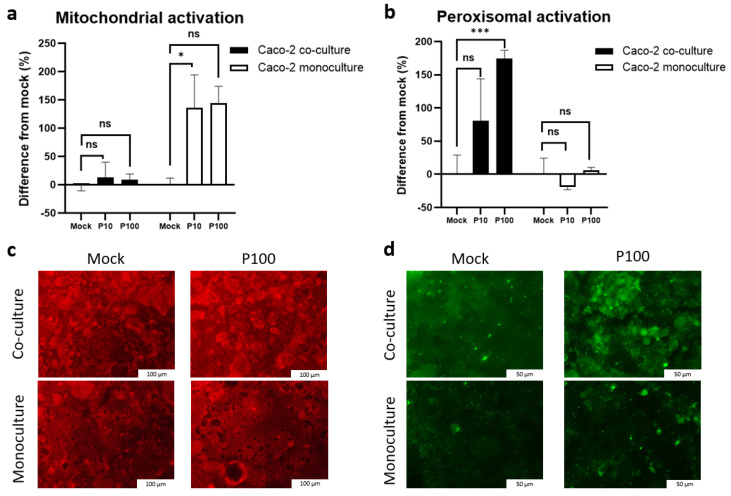
Mitochondrial and peroxisomal activation in the co-culture compared to the monoculture assessed using immunofluorescent staining: (**a**) quantification of the intensity of mitochondrial activation, expressed as the difference from the mock-treated controls; (**b**) quantification of the intensity of peroxisomal activation, expressed as the difference from the mock-treated controls; (**c**) representative immunofluorescence images of mitochondrial activation detected through MitoTracker staining; (**d**) representative immunofluorescence images showing peroxisomal activation detected through PEX-1 staining. ns indicates *p* > 0.05, * indicates *p* < 0.05, and *** indicates *p* < 0.001.

To examine the antiviral mechanisms further, we assessed the organelle-specific activation, focusing on the mitochondria and peroxisomes due to their key roles in innate immunity. The quantitative analysis (Figure 7a,b) and fluorescence imaging (Figure 7c,d) revealed distinct activation patterns between the models.

*Mitochondrial activation* (Figure 7a,c): In the monoculture, Poly I:C triggered an up to 200% increase in mitochondrial activity (9.32 ± 0.9 a.u. in mock vs. 23.1 ± 4.46 a.u. and 21.86 ± 2.27 a.u. after 10 and 100 µg/mL of Poly I:C, *p* = 0.01 and *p* = 0.008, respectively) (Figure 7a). In contrast, the co-culture exhibited higher baseline mitochondrial activation (29.57 ± 2.1 a.u.) with no significant change after stimulation, suggesting reduced reliance on mitochondrial antiviral signaling pathways (Figure 7a).

*Peroxisomal activation* (Figure 7b,d): Conversely, the co-culture exhibited a strong, dose-dependent increase in peroxisomal activity (80–175% over the mock) (Figure 7b,d), whereas the monocultures showed negligible effects or even slightly reduced activation (Figure 7b,d).

Peroxisomal activation was strongly correlated with IRF3 expression (r = 0.894 at 4 h in all cultures; r = 0.949 at 4 h in the co-culture), further supporting its functional relevance. TBK1 and TRIF levels also showed strong correlations with the peroxisomal activity (r = 0.949, *p* = 0.002 and r = 0.947, *p* = 0.002, respectively), which was comparatively lower in the monocultures. These findings suggest that co-culture conditions promote peroxisomal MAVS-TBK1-IRF3 antiviral signaling, potentially providing a more controlled and less cytotoxic alternative to the mitochondria-driven pathways, aligning with the sustained TBK1 expression and reduced levels of apoptosis observed in the co-culture conditions.

### 3.8. STAT3 Signaling Across Models Does Not Mirror TBK1/IFNLR1 Patterns

**Figure 8:**
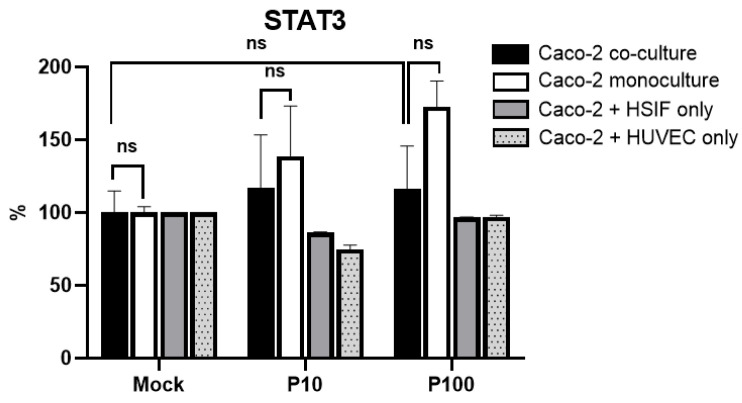
Comparison of STAT3 expression using ELISA in co-culture and monoculture cells 24 h after Poly I:C stimulation. ns indicates *p* > 0.05.

The analysis of the phosphorylated STAT3 (Tyr705) levels revealed a different pattern. The Caco-2 monocultures showed an increase in STAT3 expression, reaching 172.79 ± 12.5% of the mock levels after high-dose Poly I:C (Figure 8). In contrast, the STAT3 levels in the triple co-cultures remained near the baseline. This suggests that STAT3 activation is enhanced in epithelial monocultures, potentially reflecting a compensatory or stress- related response, rather than a coordinated antiviral signaling mechanism.

Importantly, in the monoculture, IRF1 was strongly associated with IRF3 (r = 0.691, *p* = 0.003), STAT signaling (r = 0.755, *p* < 0.001), and mitochondrial activation (r = 0.705, *p* = 0.0049). In contrast, in the co-culture, the IRF3 levels strongly correlated with IFNλ1 (r = 0.865, *p* = 0.013 at 4 h; r = 0.987, *p* < 0.001 at 24 h) and TBK1 (r = 0.999, *p* < 0.001 at 4 h; r = 0.852, *p* = 0.016 at 24 h), highlighting their coordinated antiviral role and indicating a distinct regulatory pattern in the presence of stromal cells.

### 3.9. Melatonin Mitigates Poly I:C-Induced Apoptosis and Enhances Cell Viability in GI Epithelial Models

To evaluate the protective effects of melatonin against viral-mimic-induced cytotoxicity, we assessed the apoptosis and cell viability in the GI epithelial monoculture and co-culture models treated using Poly I:C, either with melatonin pretreatment (administered 24 h before Poly I:C) or treatment (administered 24 h after Poly I:C stimulation).

**Figure 9:**
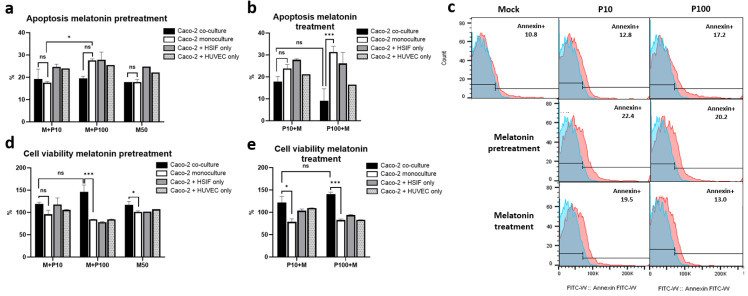
Apoptosis and cell viability comparison between co-culture and monoculture models after stimulation with Poly I:C before and after treatment with melatonin: (**a**) apoptosis in cultures when melatonin was applied before Poly I:C stimulation; (**b**) apoptosis in cultures when melatonin was applied after Poly I:C stimulation; (**c**) histograms comparing apoptosis in co-cultures in melatonin-naïve, -pretreated and -treated Caco-2 cells after stimulation with Poly I:C (blue histogram shows unstained control, red–annexin expression); (**d**) cell viability in cultures when melatonin was applied before Poly I:C stimulation; (**e**) cell viability in cultures when melatonin was applied after Poly I:C stimulation. ns indicates *p* > 0.05, * indicates *p* < 0.05, and *** indicates *p* < 0.001.

The melatonin pretreatment reduced apoptosis and preserved cell viability (Figure 9). In the monoculture, pretreatment with melatonin (50 μM for 24 h) moderately reduced apoptosis, particularly following high-dose Poly I:C (41.3 ± 4.7% in untreated cells vs. 27.65 ± 0.64% in pretreated cells, *p* = 0.001) (Figure 9a). In the co-culture, the levels of apoptosis were reduced further compared to those in the monocultures or dual co-cultures (Caco-2 + HSIFs or Caco-2 + HUVECs only), suggesting a protective effect of the multicellular environment (Figure 9a,c). Correspondingly, the cell viability was significantly higher in the co-culture under the pretreatment conditions, particularly after high-dose Poly I:C, reaching 158.7 ± 16.95% of the mock levels, indicating enhanced stress resistance (Figure 9d).

Treatment with melatonin conferred even stronger protection (Figure 9b). In the high-dose-Poly I:C-treated co-cultures, the levels of apoptosis dropped to 9.12 ± 5.49%, compared to 31.3 ± 2.69% in the monoculture (*p* < 0.001) (Figure 9b,c). Cell viability improved markedly, especially in the triple co-culture model, exceeding 150% of the mock levels after high-dose Poly I:C stimulation (Figure 9e), confirming the strong cytoprotective effect of melatonin.

### 3.10. Melatonin Maintains IFNλ1 and IFNLR1 Signaling, Especially with Pretreatment

To further understand melatonin’s modulatory effects on antiviral immunity, we investigated its impact on critical signaling molecules and organelle-specific activation in both the pre- and post-Poly I:C treatment settings across the monoculture and co-culture models.

**Figure 10:**
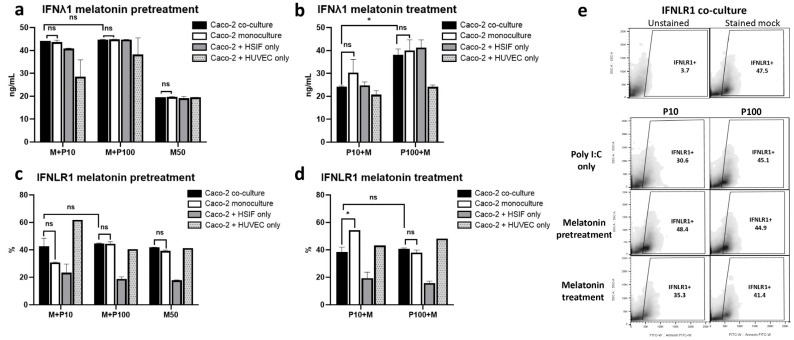
The levels of IFNλ1 quantified through ELISA at 24 h in the Poly I:C-stimulated melatonin-pretreated (**a**) and -treated (**b**) cells. The IFNLR1 expression assessed through flow cytometry at 24 h in the Poly I:C-stimulated melatonin-pretreated (**c**) and -treated (**d**) cells. (**e**) Density plots showing the effect of melatonin on the expression of IFNLR1 in Caco-2 cells grown in co-culture with HSIFs and HUVECs and treated with 50 μM of melatonin before and after Poly I:C stimulation in comparison to that in stained and unstained control non-infected cells (*n* = 3). ns indicates *p* > 0.05, * indicates *p* < 0.05.

As shown in Figure 10a, melatonin pretreatment sustained high IFNλ1 levels across all models (around 45 ng/mL; *p* = 0.254), indicating that melatonin does not suppress, and may even enhance, antiviral cytokine production when administered prior to viral stimulation. In contrast, post-treatment led to a modest reduction in IFNλ1 (20–40 ng/mL), with a tendency towards a stronger effect in the co-culture, though its levels remained within a functional antiviral range (Figure 10b), potentially reflecting feedback regulation in the protected state.

The IFNLR1 expression followed a similar trend (Figure 10c-e). In the co-culture, the same levels of this receptor (40–45%) were maintained under both the pre- and post-treatment conditions, while the monoculture and the Caco-2 + HSIF model exhibited greater downregulation in the IFNLR1 expression, particularly after treatment following low-dose Poly I:C stimulation (Figure 10c,d). These results suggest that melatonin helps maintain IFN responsiveness, with co-cultures being more resilient to receptor downregulation under immune stress.

### 3.11. Melatonin Represses IRF1 and IRF3 Signaling Preferentially in Co-Cultures

**Figure 11:**
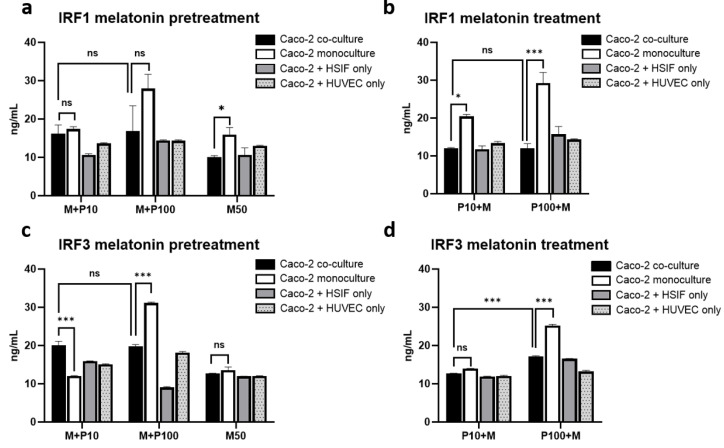
Levels of IRFs assessed through ELISA in co-culture and monoculture models when melatonin was applied before and after stimulation with Poly I:C: levels of IRF1 4 h after Poly I:C stimulation in melatonin-pretreated (**a**) and melatonin-treated (**b**) cells; levels of IRF3 4 h after Poly I:C stimulation in melatonin-pretreated (**c**) and melatonin-treated (**d**) cells. ns indicates *p* > 0.05, * indicates *p* < 0.05, and *** indicates *p* < 0.001.

Melatonin modulated the expression of key transcription factors involved in antiviral responses, with the effects varying by the model. The IRF1 expression was only modestly reduced by pretreatment (Figure 11a), but the post-treatment significantly suppressed IRF1 in the co-culture (Figure 11b), suggesting that melatonin helps to limit excessive IRF1-driven inflammatory signaling under stress conditions.

Melatonin also reduced IRF3 most noticeably in the co-culture compared with its effect in the other models (*p* < 0.01). The IRF3 levels in the co-cultures were more strongly suppressed under the post-treatment conditions, particularly in response to high-dose Poly I:C (Figure 11c,d). Despite this reduction, functional antiviral signaling was maintained, supporting the notion that melatonin fine-tunes the IRF3–IFNλ1 axis rather than fully inhibiting it.

### 3.12. STAT3 Activation Is Attenuated by Melatonin in Co-Cultures but Elevated in Monocultures

**Figure 12:**
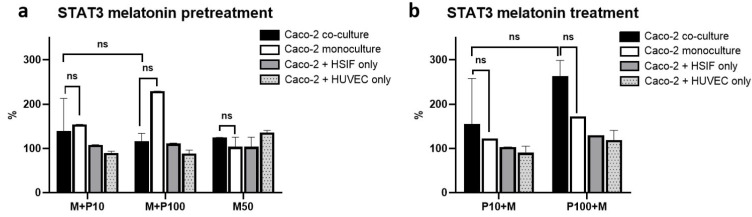
A comparison of the levels of STAT3 quantified through ELISA in the co-culture and monoculture models when melatonin was applied before (**a**) and after (**b**) stimulation with Poly I:C. ns indicates *p* > 0.05.

Melatonin exerted opposing effects on STAT3 signaling across the models (*p* = 0.004). In the monoculture, melatonin pretreatment resulted in a pronounced increase in the STAT3 activity, especially following high-dose Poly I:C (185.3 ± 12.5% in untreated cells vs. 229 ± 0.2% of the mock levels in pretreated cells, *p* = 0.046) (Figure 12). In contrast, in the co-cultures, the STAT3 activation remained moderate regardless of the treatment. This finding supports the concept that melatonin helps to prevent pro-inflammatory overactivation of STAT3 in a multicellular environment, contributing to a more balanced antiviral response.

### 3.13. Melatonin Enhances the TBK1 Expression Specifically in Co-Cultures Without Altering TRIF Levels

**Figure 13:**
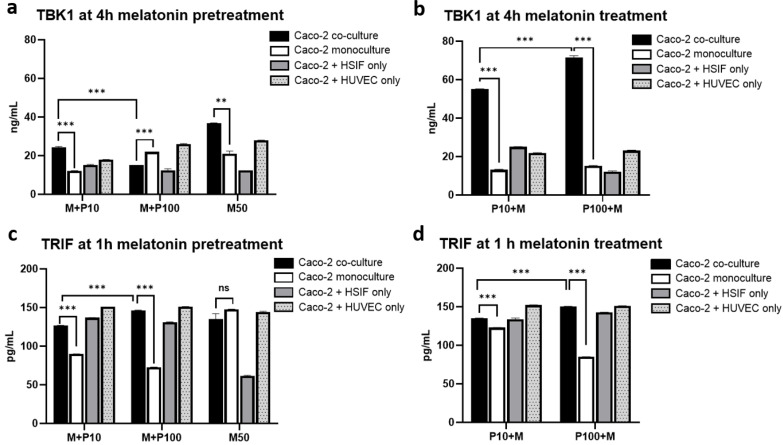
A comparison of the levels of TBK1 and TRIF quantified through ELISA in the co-culture and monoculture models when melatonin was applied before and after stimulation with Poly I:C: (**a**) levels of TBK1 in the melatonin-pretreated cells 4 h after Poly I:C stimulation; (**b**) levels of TBK1 in the melatonin-treated cells 4 h after Poly I:C stimulation; (**c**) levels of TRIF in the melatonin-pretreated cells 1 h after Poly I:C stimulation; (**d**) levels of TBK1 in the melatonin-treated cells 1 h after Poly I:C stimulation. ns indicates *p* > 0.05, ** indicates *p* < 0.01, and *** indicates *p* < 0.001.

As shown in Figure 13b, the effect of melatonin on TBK1 levels depended on the culture model (*p* < 0.0001). The TBK1 expression was significantly upregulated in the triple co-culture following melatonin treatment, particularly after high-dose Poly I:C stimulation (49.2 ± 0.28 ng/mL in untreated cells vs. 71.5 ± 0.96 ng/mL melatonin-treated cells), but not in the monocultures or dual co-cultures (Figure 13a,b). In contrast, the TRIF levels remained relatively unchanged under all conditions in the triple co-culture (Figure 13c,d), suggesting that melatonin acts downstream of or independently from TRIF. These findings point toward the possible post-transcriptional regulation of TBK1 or its activation via upstream stress-related mechanisms—potentially involving peroxisomal signaling, as discussed below.

### 3.14. Melatonin Modulates Organelle Activation

**Figure 14:**
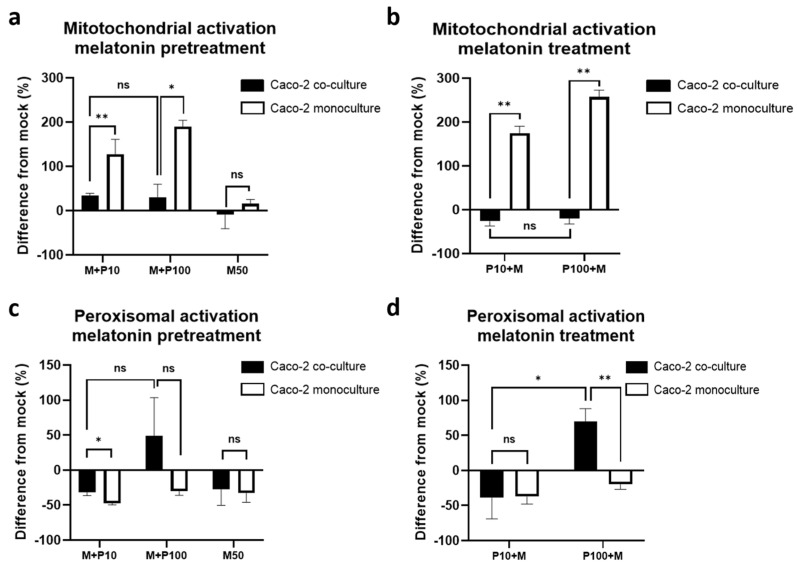
The effect of melatonin on mitochondrial and peroxisomal activation assessed through immunofluorescent staining in the co-culture compared to that in the monoculture. Quantification of the intensity of mitochondrial activation, expressed as the difference from the mock-treated controls when melatonin was applied before (pretreatment) (**a**) stimulation with Poly I:C or after (treatment) (**b**); quantification of the intensity of mitochondrial activation, expressed as the difference from the mock-treated controls when melatonin was applied before (pretreatment) (**c**) stimulation with Poly I:C or after (treatment) (**d**). ns indicates *p* > 0.05, * indicates *p* < 0.05, ** indicates *p* < 0.01.

Melatonin altered the organelle-specific activation patterns in a model- and timing-dependent manner (Figure 14). In the monocultures, melatonin further amplified the Poly I:C-induced mitochondrial activation, particularly after high-dose Poly I:C stimulation (Figure 14a,b), potentially enhancing oxidative stress. However, this was not observed in the co-culture, where melatonin reduced or stabilized mitochondrial activation, aligning with its observed anti-apoptotic effects.

Peroxisomal activation was reduced under both pre- and post-treatment conditions in both culture models (*p* = 0.002), with a clearer effect observed in the co-culture (Figure 14c,d). The organelle shift from peroxisome-driven antiviral signaling might reflect a regulatory mechanism through which melatonin promotes TBK1–IRF3–IFNλ1 axis activation in co-cultures while avoiding mitochondrial stress pathways that are more prominent in monocultures.

## 4. Discussion

**Figure 15:**
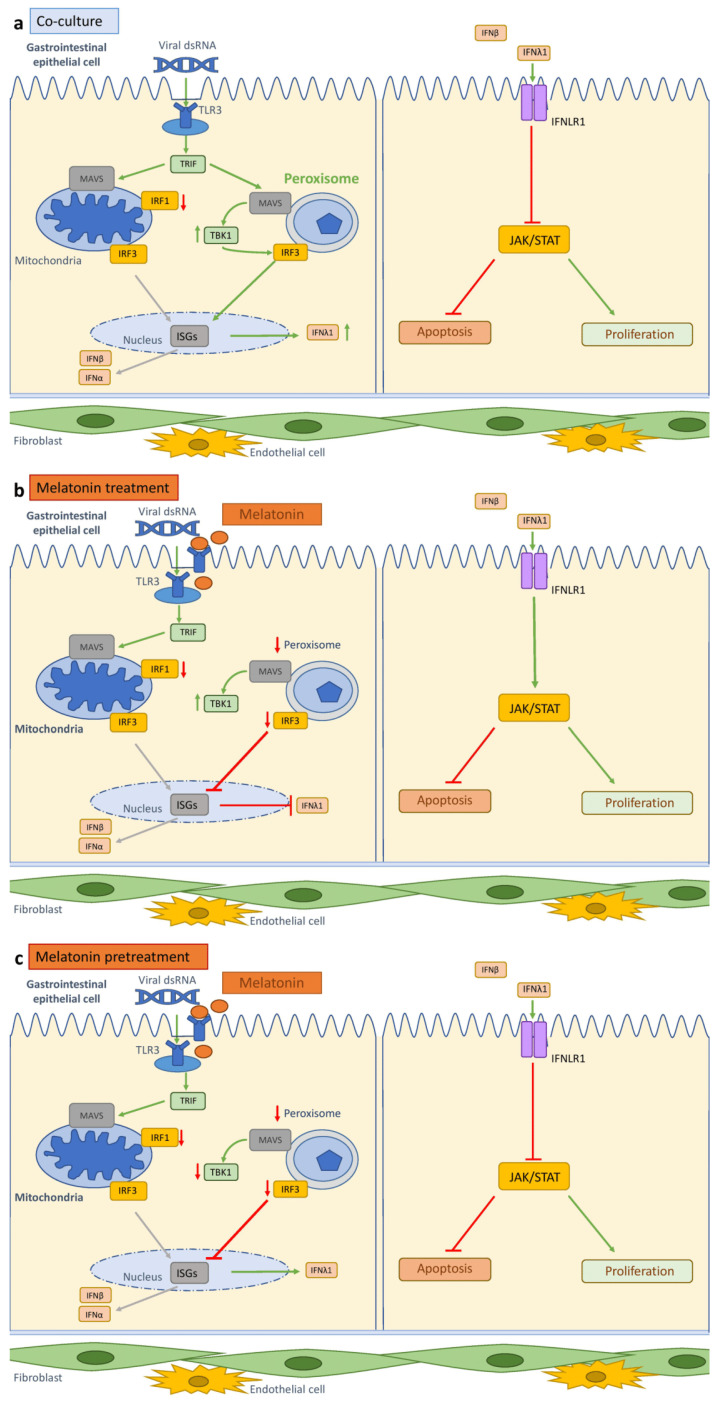
A schematic summary of the study results: (**a**) the Caco-2 cells cultured with HSIFs and HUVECs, compared to the monoculture, demonstrated enhanced peroxisomal activation, higher TBK1 expression, and a stronger and more rapid IRF3 response which resulted in reduced apoptosis; (**b**) melatonin treatment in the Caco-2 cells cultured with HSIFs and HUVECs modulated organelle-specific antiviral signaling by suppressing peroxisomal activation and stabilizing mitochondrial activity, which resulted in reduced TBK1, IRF3, and IFNλ1 levels and modulated STAT3 signaling, as well as further reductions in apoptosis; (**c**) melatonin pretreatment in Caco-2 cells cultured with HSIFs and HUVECs modulated antiviral signaling by suppressing peroxisomal activation and promoting mitochondrial activity, which resulted in reduced TBK1, IRF1, and IRF3 levels, as well as further reductions in apoptosis.

In this study, we developed a physiologically relevant GI co-culture model incorporating epithelial (Caco-2) cells, fibroblasts (HSIFs), and endothelial cells (HUVECs) to investigate the antiviral responses and their modulation by melatonin. Our findings demonstrate that the multicellular architecture of this model not only enhances epithelial growth and viability but also confers resistance to virus-mimic-induced cytotoxicity through the coordinated activation of type III interferon signaling and organelle-specific innate immune pathways (Figure 15a). Furthermore, melatonin significantly amplified these protective effects, reinforcing its potential as a mucosal immunomodulator (Figure 15b,c).

Compared to the Caco-2 monoculture, the co-culture model exhibited increased cell proliferation and markedly reduced Poly I:C-induced apoptosis, suggesting that fibroblasts and endothelial cells provide essential trophic and protective support to the intestinal epithelium. This protection was evidenced further by the preserved cell viability and reduced cytotoxic stress under high-dose viral mimic conditions. These results align with growing recognition of the role of the stromal cells in maintaining epithelial integrity and shaping mucosal immune responses, which could be mediated by paracrine factors, such as growth factors or cytokines, direct cell–cell interactions, or increased cellular polarization [6,15,16,17].

A key antiviral feature of the co-culture system was the enhanced induction of type III interferon (IFNλ1), a cytokine class known to be critical in mucosal surfaces, including the gut epithelium [18,19,20]. The co-cultures not only induced IFNλ1 earlier than the monocultures but also maintained the IFNLR1 expression even after immune challenge, supporting the potential for prolonged epithelial responsiveness. Notably, IFNα was undetectable in all models, and IFNβ was only modestly upregulated, consistent with a compartmentalized IFN response favoring type III interferons in the epithelial tissues [18,19,20,21]. This selective activation of type III IFNs in the intestinal mucosa helps to preserve the mucosal barrier, ensuring a balance between protective and detrimental antiviral responses without eliciting the systemic inflammatory effects associated with type I IFNs [19,22,23].

At the transcriptional level, in the co-culture, we observed a distinct interferon regulatory pattern: IRF3 was rapidly and strongly induced, correlating with the IFNλ1 and TBK1 levels, whereas IRF1 was significantly more elevated in the monoculture. This suggests that the stromal cells help modulate inflammatory signaling by promoting IRF3-driven antiviral signaling while suppressing IRF1 activation. Although IRF1 contributes to the expression of interferon-stimulated genes (ISGs) in the intestinal epithelial cells and may be necessary for IRF3 activation [22,24], we observed limited IRF1 induction in the co-culture model. Recent research suggests that IRF1 transcription is mainly induced by type I interferons [23], the expression of which was limited in our model. Moreover, our findings align with studies showing that IRF3 can still be induced after viral infection even when IRF1 is knocked down, suggesting their independent activation [22]. Although we did not assess the upstream PRR expression or RNA levels, the enhanced peroxisomal activity observed in the co-cultures may explain the differential regulation of IRF3. As peroxisome-localized MAVS is known to promote rapid, IRF3-driven responses with a lower inflammatory output compared to that for mitochondrial MAVS, this shift in organelle signaling could underlie the preferential IRF3 activation seen in the co-culture. However, additional functional assays, such as assessments of IRF3 phosphorylation and nuclear translocation, are needed to confirm its activation and mechanistic role in the antiviral response.

Several mechanisms have been proposed to explain the preferential induction of type III IFN production in the intestinal epithelial cells, including high IFNLR1 expression, mucosal-specific co-factors, and the activation of MAVS localized in highly abundant peroxisomes [18,19]. In recent years, studies have highlighted the crucial role of the peroxisomes in innate antiviral responses in the gut [22,24,25]. Odendall et al. [22] demonstrated that type III IFN production could be boosted by an increased number of peroxisomes upon differentiation of the intestinal epithelial cells without any effect on the type I IFN response. Although we did not quantify peroxisomal abundance directly, our immunofluorescence data revealed increased peroxisomal activation in the co-cultured Caco-2 cells following viral mimic stimulation, strongly correlating with IRF3 levels. While IRF3 can also be activated in the mitochondria, given that peroxisomal MAVS triggers faster IFN responses than mitochondrial MAVS [24], this may explain the accelerated IFNλ1 production in the co-cultures. Importantly, this effect was less pronounced in the dual co-cultures, suggesting that the enhanced peroxisomal role observed in the co-cultured cells cannot be attributed solely to epithelial differentiation and that synergistic interactions among multiple stromal cell types are important. Additionally, peroxisomes can be transferred through the culture medium and activate antiviral responses in other cells [22], potentially impacting epithelial cell responses.

TBK1, a key regulator downstream of MAVS, exhibited higher baseline and sustained levels following Poly I:C stimulation in the co-culture model compared to those in the monoculture, indicating a primed antiviral state. It also highly correlated with enhanced peroxisomal activation, suggesting preferential use of the peroxisomal MAVS–TBK1 axis over mitochondrial signaling. Peroxisomal-mediated responses are known to be robust yet less inflammatory, particularly suited to mucosal defense by limiting collateral damage [24]. Although TBK1 is known to act downstream of MAVS in both organelles [24], its functional link to peroxisomal activation, to our knowledge, has not been directly characterized, highlighting a novel aspect of mucosal antiviral regulation. While our findings suggest a shift toward peroxisome-dominant antiviral signaling in co-cultures, the MAVS localization was not directly assessed. We used peroxisomal and mitochondrial markers to evaluate organelle activity, but this did not confirm the compartment-specific MAVS activation. Further studies are needed to validate whether the observed effects are mediated through MAVS signaling.

Melatonin, a pleiotropic indoleamine produced by the pineal gland and extra-pineal sites including the GI tract, has well-established antioxidant, anti-inflammatory, and immunomodulatory properties [26,27,28]. Here, we have demonstrated that melatonin administration, particularly after Poly I:C stimulation, markedly reduced apoptosis and preserved cell viability in the intestinal epithelial models, with the most pronounced effects observed in the triple co-culture system. This finding aligns with previous studies in which melatonin was shown to protect the intestinal epithelium from oxidative stress, inflammatory injury, and viral challenge [12,29].

Importantly, melatonin’s cytoprotective effects did not occur at the expense of antiviral signaling. On the contrary, melatonin pretreatment maintained high levels of IFNλ1, suggesting that it supports, or even enhances, mucosal antiviral immunity. This was further corroborated by the sustained expression of IFNLR1 in the co-culture models, ensuring functional responsiveness to type III IFNs. In contrast to broad-spectrum immunosuppressants, melatonin appears to fine-tune the balance between immune activation and epithelial integrity.

We observed that melatonin modulated the expression of key transcription factors, particularly IRF3 and IRF1. The IRF1 and IRF3 expression was significantly suppressed, especially in the post-treatment setting. Given that excessive IRF1 activity has been associated with heightened inflammatory signaling and tissue damage [23], its downregulation in the co-cultures likely contributed to the controlled antiviral response observed here. Similarly, melatonin also stabilized STAT3 activation, particularly in the co-cultures, while the monocultures exhibited exaggerated STAT3 signaling. Since STAT3 plays dual roles in inflammation and epithelial proliferation [30], its overactivation may have led to dysregulated immune responses in the monoculture, highlighting the importance of the multicellular context to interpreting the immune dynamics. However, the total STAT3 protein levels were not measured, which limited the ability to distinguish whether the observed changes reflect increased phosphorylation or an altered overall expression.

A unique and novel aspect of our findings is the observation that melatonin redirected innate immune signaling away from the peroxisomes and toward a more balanced mitochondrial pathway under co-culture conditions. While the monocultures exhibited a sharp increase in mitochondrial activity (and possibly oxidative stress) after the Poly I:C stimulation and melatonin treatment, the co-cultures maintained stable mitochondrial activation and instead displayed reduced but functionally relevant peroxisomal activity. Given that peroxisomes are increasingly recognized as signaling hubs for antiviral responses with reduced inflammatory potential [22,24], this shift may explain the lower apoptosis levels and more controlled IFN response in the melatonin-treated co-cultures.

The peroxisome-dependent signaling pathway appeared to converge for TBK1, whose expression was significantly enhanced in the co-cultures after melatonin treatment, while the TRIF levels remained largely unchanged. This suggests that melatonin may post-transcriptionally regulate TBK1 or enhance the peroxisomal MAVS activity upstream—resulting in robust IRF3-mediated IFNλ1 production without exacerbating pro-inflammatory signaling cascades. These findings point to a previously underexplored mechanism through which melatonin modulates antiviral immunity at the subcellular level, particularly relevant to gut epithelial defense, though further validation using functional inhibition strategies (e.g., TBK1 or IRF3 silencing) is warranted to confirm the causality.

The GI tract is both a major site of viral entry and replication and a major source of endogenous melatonin, produced locally by the enterochromaffin cells [31]. This raises intriguing physiological implications: endogenous melatonin may serve as a homeostatic modulator of antiviral defenses in the gut, particularly under stress or infection. Furthermore, melatonin levels decline with age and in chronic disease states [32], potentially compromising mucosal immunity. Our findings suggest that restoring or boosting melatonin levels—through supplementation or lifestyle interventions—could enhance mucosal resilience, especially during enteric viral infections or in vulnerable populations.

Melatonin’s safety profile, low cost, and pleiotropic benefits have spurred interest in its therapeutic repurposing, particularly in the context of respiratory and viral GI infections [10,33,34,35]. The current study extends this rationale to gut models, suggesting that melatonin could function as a valuable adjunct in managing intestinal viral pathologies, especially when combined with strategies that preserve or mimic the multicellular mucosal architecture.

Our study has some additional limitations. Firstly, we used Caco-2 cells, which are of an adenocarcinoma origin and might exhibit different signaling pathways compared to those in healthy GI epithelial cells. Secondly, while our model revealed that stromal cells play an important role in epithelial antiviral responses and suggested a possible central role of the peroxisomes, the pathways may differ in vivo in the presence of immunomodulatory cells. Thirdly, we relied solely on immunofluorescence data to assess the peroxisomal activity, which may not have fully captured the extent of the functional consequences of organelle signaling; thus, further studies employing additional methodologies are needed. Lastly, while our findings suggest that melatonin modulates organelle-specific antiviral signaling, the precise mechanisms remain unclear. Future research should aim to elucidate how melatonin influences peroxisomal and mitochondrial pathways in the context of antiviral responses.

## 5. Conclusions

Our findings demonstrate that the co-presence of fibroblast and endothelial cells not only enhances epithelial growth and viability but also confers resistance to virus-mimic-induced cytotoxicity. Our results align with those of previous studies demonstrating that melatonin enhances the TBK1–IRF3 axis while suppressing IRF1 and STAT3 activation—mechanisms that favor type III interferon production while limiting inflammation. Additionally, we show that melatonin modulates these antiviral responses by downregulating peroxisomal activation and promoting mitochondrial signaling, suggesting an organelle-specific mechanism of action. Our findings help fill the gap in our understanding of melatonin’s role in the antiviral response in the intestinal mucosa. However, further studies are needed to investigate the pathways through which the stromal cells and melatonin coordinate organelle-specific antiviral signaling in greater detail.

## Figures and Tables

**Table 1:** Composition of culture media for different cell types used in co-culture models.

Cells	Medium Composition
**Caco-2**	MEM+ 10% FBS (Gibco, Life Technologies NZ Ltd., Auckland, New Zealand) + 1% NEAAs (Gibco, Life Technologies Limited, Paisley, UK) + 1% P/S (Gibco, Life Technologies Limited, Paisley, UK)
**HSIF**	DMEM (Gibco, Life Technologies Limited, Paisley, UK) + 10% FBS + 1% P/S
**HUVEC**	HLVEC Basal Medium (Gibco, Life Technologies Corp., Grand Island, NY, USA) + 10% FBS + 1% P/S + LSGS (Gibco; Cascade Biologics; Life Technologies Corp., Grand Island, NY, USA)

**Abbreviations:** MEM—Minimum Essential Medium; FBS—Fetal Bovine Serum; NEAAs—Non-Essential Amino Acids; P/S—Penicillin/Streptomycin; DMEM—Dulbecco’s Modified Eagle Medium; HLVEC—Human Large Vessel Endothelial Cell; LSGS—Low Serum Growth Supplement.

**Table 2:** The analytes measured using the ELISA and their detection limits.

Analyte and Manufacturer	Detection Limit
IFNλ1 (Abcam, Cambridge, UK)	13.72 ng/mL
IFNβ (Abcam, Cambridge, UK)	9.38 pg/mL
IFNα (Invitrogen, Bender MedSystems GmbH, Vienna, Austria)	7.8 pg/mL
phospho-STAT3 (Tyr705; Invitrogen, Thermo Fisher Scientific, Life Technologies Corp., Carlsbad, CA, USA)	Semi-quantitative
IRF1 (ELK Biotechnology, Denver, USA)	0.32 ng/mL
IRF3 (ELK Biotechnology, Denver, USA)	0.16 ng/mL
TRIF (ELK Biotechnology, Denver, USA)	78.13 pg/mL
TBK1 (ELK Biotechnology, Denver, USA)	0.32 ng/mL

## Data Availability

The datasets presented in this article are not readily available because the data are part of an ongoing project. Requests to access the datasets should be directed to the corresponding author.

## Abbreviations

The following abbreviations are used in this manuscript:

Caco-2	Human Epithelial Colorectal Adenocarcinoma Cell Line
CCK-8	Cell Counting Kit-8
CLD	Compact Letter Display
DMEM	Dulbecco’s Modified Eagle Medium
ELISA	Enzyme-Linked Immunosorbent Assay
FBS	Fetal Bovine Serum
GI	Gastrointestinal Tract
HSIF	Human Small Intestinal Fibroblast
HUVEC	Human Umbilical Vein Endothelial Cell
IFN	Interferon
IFNα	Interferon Alpha
IFNβ	Interferon Beta
IFNλ1	Interferon Lambda 1
IFNLR1	Interferon Lambda Receptor 1
IRF1	Interferon Regulatory Factor 1
IRF3	Interferon Regulatory Factor 3
JAK/STAT	Janus Kinase/Signal Transducer and Activator of Transcription
LSGS	Low Serum Growth Supplement
MEM	Minimum Essential Medium
MFI	Median Fluorescence Intensity
NEAA	Non-Essential Amino Acid
PBS	Phosphate-Buffered Saline
PFA	Paraformaldehyde
PI	Propidium Iodide
Poly I:C	Polyinosinic–Polycytidylic Acid
P/S	Penicillin/Streptomycin
ROI	Region of Interest
SMA	Smooth Muscle Actin
STAT3	Signal Transducer and Activator of Transcription 3
TBK1	TANK-Binding Kinase 1
TLR3	Toll-Like Receptor 3
TRIF	TIR-Domain-Containing Adapter-Inducing Interferon-β

## References

[1] Neurath M.F., Artis D., Becker C. (2025). The intestinal barrier: A pivotal role in health, inflammation, and cancer. Lancet Gastroenterol. Hepatol..

[2] Julio-Pieper M., López-Aguilera A., Eyzaguirre-Velásquez J., Olavarría-Ramírez L., Ibacache-Quiroga C., Bravo J.A., Cruz G. (2021). Gut Susceptibility to Viral Invasion: Contributing Roles of Diet, Microbiota and Enteric Nervous System to Mucosal Barrier Preservation. Int. J. Mol. Sci..

[3] Wang J., Li H., Xue B., Deng R., Huang X., Xu Y., Chen S., Tian R., Wang X., Xun Z. (2020). IRF1 Promotes the Innate Immune Response to Viral Infection by Enhancing the Activation of IRF3. J. Virol..

[4] Pervolaraki K., Talemi S.R., Albrecht D., Bormann F., Bamford C., Mendoza J.L., Garcia K.C., McLauchlan J., Höfer T., Stanifer M.L. (2018). Differential induction of interferon stimulated genes between type I and type III interferons is independent of interferon receptor abundance. PLoS Pathog..

[5] Perry A.K., Chow E.K., Goodnough J.B., Yeh W.C., Cheng G. (2004). Differential requirement for TANK-binding kinase-1 in type I interferon responses to toll-like receptor activation and viral infection. J. Exp. Med..

[6] Darling N.J., Mobbs C.L., González-Hau A.L., Freer M., Przyborski S. (2020). Bioengineering Novel in vitro Co-culture Models That Represent the Human Intestinal Mucosa With Improved Caco-2 Structure and Barrier Function. Front. Bioeng. Biotechnol..

[7] Su R., Shereen M.A., Zeng X., Liang Y., Li W., Ruan Z., Li Y., Liu W., Liu Y., Wu K. (2020). The TLR3/IRF1/Type III IFN Axis Facilitates Antiviral Responses against Enterovirus Infections in the Intestine. mBio.

[8] Riemann D., Espie C.A., Altena E., Arnardottir E.S., Baglioni C., Bassetti C.L.A., Bastien C., Berzina N., Bjorvatn B., Dikeos D. (2023). The European Insomnia Guideline: An update on the diagnosis and treatment of insomnia 2023. J. Sleep Res..

[9] Kitidee K., Samutpong A., Pakpian N., Wisitponchai T., Govitrapong P., Reiter R.J., Wongchitrat P. (2023). Antiviral effect of melatonin on Japanese encephalitis virus infection involves inhibition of neuronal apoptosis and neuroinflammation in SH-SY5Y cells. Sci. Rep..

[10] Lempesis I.G., Georgakopoulou V.E., Reiter R.J., Spandidos D.A. (2024). A mid pandemic night’s dream: Melatonin, from harbinger of anti-inflammation to mitochondrial savior in acute and long COVID-19 (Review). Int. J. Mol. Med..

[11] Hardeland R. (2018). Melatonin and inflammation—Story of a double-edged blade. J. Pineal Res..

[12] Šeškutė M., Žukaitė D., Laucaitytė G., Inčiūraitė R., Malinauskas M., Jankauskaitė L. (2024). Antiviral Effect of Melatonin on Caco-2 Cell Organoid Culture: Trick or Treat?. Int. J. Mol. Sci..

[13] Bubenik G.A. (2002). Gastrointestinal melatonin: Localization, function, and clinical relevance. Dig. Dis. Sci..

[14] Kim S.W., Kim S., Son M., Cheon J.H., Park Y.S. (2020). Melatonin controls microbiota in colitis by goblet cell differentiation and antimicrobial peptide production through Toll-like receptor 4 signalling. Sci. Rep..

[15] Bhushal S., Wolfsmüller M., Selvakumar T.A., Kemper L., Wirth D., Hornef M.W., Hauser H., Köster M. (2017). Cell Polarization and Epigenetic Status Shape the Heterogeneous Response to Type III Interferons in Intestinal Epithelial Cells. Front. Immunol..

[16] Kayama H., Takeda K. (2024). Regulation of intestinal epithelial homeostasis by mesenchymal cells. Inflamm. Regen..

[17] Eenjes E., Grommisch D., Genander M. (2023). Functional Characterization and Visualization of Esophageal Fibroblasts Using Organoid Co-Cultures. J. Vis. Exp..

[18] Hemann E.A., Gale M., Savan R. (2017). Interferon Lambda Genetics and Biology in Regulation of Viral Control. Front. Immunol..

[19] Lazear H.M., Schoggins J.W., Diamond M.S. (2019). Shared and Distinct Functions of Type I and Type III Interferons. Immunity.

[20] Wright A.P., Nice T.J. (2024). Role of type-I and type-III interferons in gastrointestinal homeostasis and pathogenesis. Curr. Opin. Immunol..

[21] Pott J., Stockinger S. (2017). Type I and III Interferon in the Gut: Tight Balance between Host Protection and Immunopathology. Front. Immunol..

[22] Odendall C., Dixit E., Stavru F., Bierne H., Franz K.M., Durbin A.F., Boulant S., Gehrke L., Cossart P., Kagan J.C. (2014). Diverse intracellular pathogens activate type III interferon expression from peroxisomes. Nat. Immunol..

[23] Forero A., Ozarkar S., Li H., Lee C.H., Hemann E.A., Nadjsombati M.S., Hendricks M.R., So L., Green R., Roy C.N. (2019). Differential Activation of the Transcription Factor IRF1 Underlies the Distinct Immune Responses Elicited by Type I and Type III Interferons. Immunity.

[24] Dixit E., Boulant S., Zhang Y., Lee A.S., Odendall C., Shum B., Hacohen N., Chen Z.J., Whelan S.P., Fransen M. (2010). Peroxisomes are signaling platforms for antiviral innate immunity. Cell.

[25] Ferreira A.R., Marques M., Ramos B., Kagan J.C., Ribeiro D. (2022). Emerging roles of peroxisomes in viral infections. Trends Cell Biol..

[26] Tan D.X., Manchester L.C., Esteban-Zubero E., Zhou Z., Reiter R.J. (2015). Melatonin as a Potent and Inducible Endogenous Antioxidant: Synthesis and Metabolism. Molecules.

[27] Maestroni G.J. (2001). The immunotherapeutic potential of melatonin. Expert Opin. Investig. Drugs.

[28] Reiter R.J., Ma Q., Sharma R. (2020). Melatonin in Mitochondria: Mitigating Clear and Present Dangers. Physiology (Bethesda).

[29] Lei X., Xu Z., Huang L., Huang Y., Tu S., Xu L., Liu D. (2024). The potential influence of melatonin on mitochondrial quality control: A review. Front. Pharmacol..

[30] Yu H., Lee H., Herrmann A., Buettner R., Jove R. (2014). Revisiting STAT3 signalling in cancer: New and unexpected biological functions. Nat. Rev. Cancer.

[31] Pal P.K., Sarkar S., Chattopadhyay A., Tan D.X., Bandyopadhyay D. (2019). Enterochromaffin cells as the source of melatonin: Key findings and functional relevance in mammals. Melatonin Res..

[32] Pandi-Perumal S.R., Bahammam A.S., Brown G.M., Spence D.W., Bharti V.K., Kaur C., Hardeland R., Cardinali D.P. (2013). Melatonin antioxidative defense: Therapeutical implications for aging and neurodegenerative processes. Neurotox. Res..

[33] Juybari K.B., Pourhanifeh M.H., Hosseinzadeh A., Hemati K., Mehrzadi S. (2020). Melatonin potentials against viral infections including COVID-19: Current evidence and new findings. Virus Res..

[34] Maestroni G.J.M. (2024). Role of Melatonin in Viral, Bacterial and Parasitic Infections. Biomolecules.

[35] Andersen L.P., Gögenur I., Rosenberg J., Reiter R.J. (2016). The Safety of Melatonin in Humans. Clin. Drug Investig..

